# Predicting Dysglycemia in Patients with Diabetes Using Electrocardiogram

**DOI:** 10.3390/diagnostics14222489

**Published:** 2024-11-07

**Authors:** Ho-Jung Song, Ju-Hyuck Han, Sung-Pil Cho, Sung-Il Im, Yong-Suk Kim, Jong-Uk Park

**Affiliations:** 1 Department of Medical Engineering, Konyang University, 158 Gwanjeo-dong-ro, Seo-gu, Daejeon 32992, Republic of Korea; songhj6692@gmail.com (H.-J.S.); dnfwlq203@gmail.com (J.-H.H.); 2 MEZOO Co., Ltd., RM.808 200, Gieopdosi-ro, Jijeong-myeon, Wonju-si 26354, Republic of Korea; spcho@me-zoo.com; 3 Division of Cardiology, Department of Internal Medicine, Kosin University Gospel Hospital, Kosin University College of Medicine, Busan 49267, Republic of Korea; sungils8932@naver.com; 4 Department of Artificial Intelligence, Konyang University, 158 Gwanjeo-dong-ro, Seo-gu, Daejeon 32992, Republic of Korea; yongsuk@konyang.ac.kr

**Keywords:** electrocardiogram, dysglycemia, heart rate variability, blood glucose, non-invasive blood glucose monitoring, artificial intelligence model

## Abstract

**Background:** In this study, we explored the potential of predicting dysglycemia in patients who need to continuously manage blood glucose levels using a non-invasive method via electrocardiography (ECG). **Methods**: The data were collected from patients with diabetes, and heart rate variability (HRV) features were extracted via ECG processing. A residual block-based one-dimensional convolution neural network model was used to predict dysglycemia. **Results:** The dysglycemia prediction results at each time point, including at the time of blood glucose measurement, 15 min prior to measurement, and 30 min prior to measurement, exhibited no significant differences compared with the blood glucose measurement values. This result confirmed that the proposed artificial intelligence model for dysglycemia prediction performed well at each time point. Additionally, to determine the optimal number of features required for predicting dysglycemia, 77 HRV features were individually eliminated in the order of decreasing importance with respect to the prediction accuracy; the optimal number of features for the model to predict dysglycemia was determined to be 12. The dysglycemia prediction results obtained 30 min prior to measurement, which exhibited the highest prediction range in this study, were as follows: accuracy = 90.5, sensitivity = 87.52, specificity = 92.74, and precision = 89.86. **Conclusions:** Furthermore, we determined that no significant differences exist in the blood glucose prediction results reported in previous studies, wherein various vital signs and blood glucose values were used as model inputs, and the results obtained in this study, wherein only ECG data were used to predict dysglycemia.

## 1. Introduction

Diabetes is an endocrine disease, primarily caused by abnormal glucose metabolism and occurring due to a combination of lifestyle, genetic, and environmental factors [1]. In particular, the risk of developing diabetes increases in middle- and old-aged people because of increased insulin resistance, aging, and lack of physical activity. Diabetes causes chronic complications that are primarily associated with vascular diseases. The most common complications include coronary artery diseases (e.g., angina and myocardial infarction), cerebrovascular diseases (e.g., stroke), and diabetic retinopathy. Additionally, diabetes can cause diabetic ketoacidosis, which is an acute complication of diabetes that may lead to death in severe cases [2]. The primary objective of diabetes management is to control blood glucose levels. Typically, the recommended range for normal blood glucose level is between 70 and 100 mg/dL for fasting blood glucose and less than 180 mg/dL for blood glucose two hours after a meal; blood glucose levels beyond these ranges are considered abnormal. Abnormal blood glucose, also referred to as dysglycemia, is categorized as hypoglycemia and hyperglycemia if the blood glucose level reduces below 70 mg/dL and exceeds 180 mg/dL, respectively [3]. Hyperglycemia is commonly observed in patients with diabetes, and studies have reported that failure to control blood glucose levels increases mortality rates and poses risks of cardiovascular diseases and neuropathy [4,5]. Conversely, impaired awareness of hypoglycemia, which refers to a diminished ability to perceive the onset of acute hypoglycemia, can lead to acute complications and even death [6,7]. Therefore, continuous management of blood glucose levels is crucial for patients experiencing dysglycemia, which includes both hyperglycemia and hypoglycemia.

Conventionally, blood glucose levels are measured using blood tests. However, this method causes significant pain and generates medical waste [8,9]. The continuous glucose monitoring system (CGMS) was developed to address these issues; this system automatically measures glucose levels in the interstitial fluid at five-to-ten-minute intervals [10]. CGMSs reduce the number of severe dysglycemic events in patients by continuously monitoring blood glucose levels and alerting them of the requirement for immediate care. Moreover, CGMSs can be combined with other measurement methods, such as insulin pumps and artificial pancreas, which are used for controlling blood glucose levels [11]. Most of the existing CGMSs employ minimally invasive methods, which reduce the pain experienced during measurements in comparison with that caused by invasive methods. However, certain issues continue to exist. For instance, miniaturized sensors may be damaged or destroyed during use, resulting in inaccurate measurements. Additionally, pressure applied to CGMSs can induce pressure-induced sensor attenuation (PISA), which not only causes pain in the tissue but can also lead to an abrupt decrease in CGMS values and an unexpected increase in the blood glucose level from the normal baseline [12,13].

To address the aforementioned issues of CGMSs, a method that utilizes blood glucose measurements and electrocardiogram (ECG) data has been proposed to predict and classify dysglycemia [14]. Several studies have determined that electrophysiological changes in the heart are associated with blood glucose levels. In particular, the results of predicting metabolic disorders, such as hyperkalemia [15], have been adequately validated. Singh et al. [16] investigated the heart rate variability (HRV) across blood glucose levels in a large population-based cohort. They analyzed the correlation between dysglycemia and HRV in 1919 people and reported that HRV, including the low-to-high-frequency (LF/HF) ratio, was reduced in patients with dysglycemia. Tobore et al. [17] attempted to minimize or eliminate the drawbacks of conventional blood glucose monitoring methods by employing a non-invasive method of measuring blood glucose levels using ECG measurements. They collected ECG data from 16 participants and measured the changes in ECG related to blood glucose; the results confirmed that changes in heart rate and HRV during glucose intake were recorded at 81%, indicating that these changes were significantly influenced by blood glucose. Chiu et al. [18] developed a machine learning (ML) algorithm that uses only ECG data and extracts important ECG features to classify dysglycemia. The results of their analysis indicated that the most important ECG feature for predicting dysglycemia was the R-R interval, followed by R-S, P-T, Q-R, S-T, R-T, and R-S intervals. Although their study used an artificial intelligence (AI) model to identify the ECG features that were important for classifying dysglycemia, they used only a few blood glucose data points and insufficient ECG data.

Several studies have investigated the correlation between ECG and dysglycemia. However, most studies use various vital signs as inputs, including blood glucose measurement values, in addition to ECG data for the dysglycemia prediction algorithm. Furthermore, dysglycemia-related studies that used only ECG data predominantly focused on the identification of dysglycemia rather than its prediction. Some studies reported models with poor performance, which are not yet suitable for clinical applications [19]. Ling et al. [20] explored a non-invasive method to identify dysglycemia by using ECG data collected from 16 patients. However, the scope of their study was limited to the identification of dysglycemia and did not include its prediction. Zhu et al. [21] aimed to develop a smartphone-based platform for predicting and managing dysglycemia using an AI model. Although the aforementioned study confirmed that dysglycemia can be predicted up to 60 min in advance, it did not demonstrate that dysglycemia can be predicted using only ECG data because blood glucose measurements, EDA, acceleration, and skin temperature were used as inputs in addition to ECG data.

In this study, we use ECG data and AI models to address various issues observed in conventional blood monitoring methods when measuring blood glucose levels. Furthermore, we deviate from previous studies that utilize multiple vital signs as inputs and rely only on ECG data. Our research focuses on predicting dysglycemia rather than only identifying the condition. Therefore, the objective of this study is not only to solve the issues observed in conventional monitoring methods but also to verify the possibility of predicting dysglycemia based on only ECG data using an AI model.

## 2. Data Collection and Selection

The data used for predicting dysglycemia were collected from 83 patients with diabetes (mean age 65.5 ± 6.2 years) who were tested for the use of medication and subjected to medical condition diagnosis in the endocrinology outpatient department at Kosin University Gospel Hospital between October 2021 and December 2021 [22].

The research protocol approval and the requirement for prior consent from individual patients were approved by the Ethics Committee of Kosin University Gospel Hospital (No. IRB 2022-06-016). We obtained written consent from individual patients, and the study was conducted in accordance with the latest version (2013) of the Helsinki Declaration principles.

We used a wearable ECG monitoring patch (Hicardi^®^; Wonju-si, Gangwon-do, Republic of Korea, MEZOO Co., Ltd., Wonju-si, Gangwon-do, Republic of Korea) certified by the Ministry of Food and Drug Safety to collect ECG data. The wearable patch monitored and recorded single-lead ECG, respiration, skin surface temperature, and activity. The ECG signals were recorded at a sampling frequency of 250 Hz with a resolution of 14 bits. Data from the wearable patch were transmitted to the application mobile gateway of a smartphone via Bluetooth Low Energy and then to a cloud-based monitoring server. After obtaining prior consent from patients, the wearable patch was attached to their left sternal border to collect data.

The participants included patients with only type 2 diabetes who were 18 years or older. Major exclusion criteria included conditions such as pregnancy, neurological disorders, heart failure, chronic liver or renal failure that could affect HRV parameters, atrial fibrillation, uncontrolled diabetes mellitus (DM), and thyroid disorders. Additionally, data from patients who failed to comply with follow-up observations or exhibited incomplete measurement monitoring were excluded from the analysis. Moreover, the ECG data of patients were selected considering signal quality; data with severe noise, unstable ECG measurements, and other data that were difficult to use for the study were excluded.

**Figure 1:**
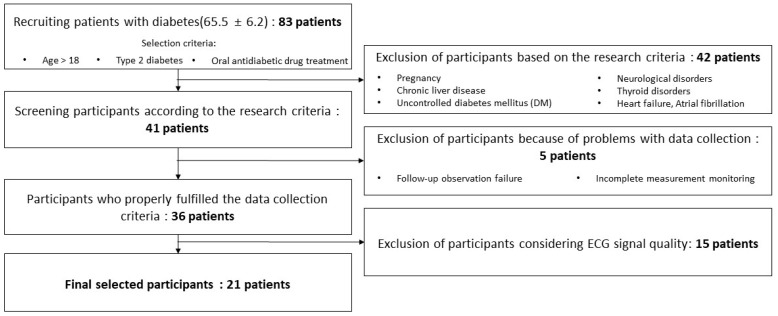
Patient data collection process.

Finally, data from 21 patients who completed ECG measurements and glucose monitoring were used in this study. Figure 1 depicts the data selection process.

We measured blood glucose levels at 15 min intervals using a glucose monitoring device to collect the blood glucose values that formed the basis for predicting dysglycemia. The measured blood glucose data comprised 42,985 values. Based on the measure values, the normal blood glucose range was defined as greater than or equal to 70 mg/dL and less than or equal to 180 mg/dL. In addition, blood glucose levels less than 70 mg/dL represented hypoglycemia, whereas blood glucose levels exceeding 180 mg/dL indicated hyperglycemia. We determined that of the 42,985 measured blood glucose data points, 26,796 and 16,189 data points were in the normal and dysglycemia ranges, respectively.

Table 1 summarizes the patient information collected in this study. The average age of the 21 patients selected (Figure 1) was 69 ± 8.6 years; female patients outnumbered male patients. The selected individuals were all patients with diabetes, with an average HbA1c measurement value of 8.1 ± 1.26 mmol/mol. The average measurement period for ECG and blood glucose data was 9.8 ± 4.53 days, during which dysglycemia levels were observed 36.82 ± 26.36% of the time (hypoglycemia 1.74 ± 1.34%; hyperglycemia 35.74 ± 26.76%). This result indicated that hyperglycemic periods were more prevalent than hypoglycemic periods. Additionally, the average measured blood glucose level was 172.14 ± 51.65 mg/dL, and the average normal blood glucose level was 128.75 ± 14.93 mg/dL. The average blood glucose levels were 64.24 ± 2.08 mg/dL and 237.03 ± 34.19 mg/dL for hypoglycemia and hyperglycemia, respectively. Therefore, both dysglycemia and normal blood glucose levels were within the defined blood glucose ranges. Additionally, there were 13 patients suffering from hypertension, and 1 patient each suffering from hyperlipidemia, fatty liver, and gastric cancer. Patients taking drugs to control diabetes include six patients prescribed insulin, eight patients prescribed metformin, and five patients prescribed SGLT2 inhibitor.

We selected ECG data collected one hour prior to measurement as the input for the AI model. Table 2 lists the ECG data selected based on the blood glucose measurement values.

A total of 2624 data points were selected. Among them, 1520 data points corresponded to normal blood glucose, and 1104 data points corresponded to dysglycemia. In other words, the normal blood glucose data points exceeded the dysglycemic data points by approximately 400. Among the dysglycemic data points, 1042 data points corresponded to hyperglycemia, and 62 data points corresponded to hypoglycemia. This implied that hyperglycemia data accounted for a larger proportion of the data. This phenomenon was attributed to the age of the patients participating in the study and the fact that data were collected from patients with only type 2 diabetes.

The 2624 data points were divided into training and validation data in the ratio of 8:2, which were used as input for the AI model. Consequently, the data points were divided into 2100 training data points (1216 normal blood glucose data points and 884 dysglycemic data points) and 524 validation data points (304 normal blood glucose data points and 220 dysglycemic data points).

## 3. Data Extraction for Dysglycemia

HRV parameters extracted from the ECG data were used as inputs for the AI model that predicts dysglycemia using ECG data. The Pan–Tompkins algorithm is widely used in ECG studies to detect QRS complexes. This algorithm applies various filters to detect QRS complexes and is known for its outstanding performance in eliminating noise [23]. Therefore, we used the Pan–Tompkins algorithm in this study as well.

The process using the Pan–Tompkins algorithm can be summarized as follows:

1. Band-pass filter: a band-pass filter is applied to accentuate the signal of QRS complexes while minimizing noise, such as muscle noise and baseline wander.
2. Derivative filter: a derivative filter is applied to extract the information on the slope of QRS complexes.
3. Squaring: the filtered signal is squared to accentuate the QRS complexes and reduce the chance of mistaking T waves for R peaks.
4. Moving window integrator: this operation is performed to obtain information on the slope and width of QRS complexes.

In the subsequent feature extraction step, the ECG data that were extracted and processed as one-hour-long data were split into five-minute units, which is the minimum unit recommended when extracting HRV parameters. HRV is an index that indicates the variability of heart rate intervals. It is a non-invasive indicator of cardiac activity that provides information related to autonomic nervous system activity. HRV is closely related to the nervous system and responds to important bodily activities such as digestion, breathing, and sensation. It involves tracking small changes in the interval between successive heartbeats, also known as the R-R interval. Recent studies have extensively investigated the relationship between dysglycemia and HRV, considering HRV as an important feature for identifying and predicting dysglycemia.

In this study, HRV parameters were extracted using the NeuroKit2 library in Python and used as a reference [24,25,26,27]. A total of 91 features were extracted, each comprising time, frequency, and nonlinear domains.

The time-domain HRV parameters reflect the variability of the overall heart rate and are divided into parameters based on the difference between RR intervals and their standard deviation. Representative time-domain parameters include MeanNN, standard deviation of normal-to-normal RR intervals (SDNN), and root mean square of successive NN interval differences (RMSSD); a total of 25 time-domain HRV parameters were used in this study.

The frequency-domain HRV parameters were extracted into various frequency range components using fast Fourier transform or automatic regression modeling. Representative frequency-domain parameters include ultra-low frequency (ULF), normalized high frequency (HFn), and LF/HF ratio. A total of 10 frequency-domain HRV parameters were used in this study.

The nonlinear-domain HRV parameters represent the unpredictability of time series data caused by the complexity of the mechanism that regulates HRV as a nonlinear exponent. Representative nonlinear-domain parameters include the parameters derived from the Poincaré plot, complexity estimation using entropy, and fractal analysis [28], which are the Porta’s index (PI), approximate entropy (ApEn), and multifractal detrended fluctuation analysis (MFDFA), respectively; a total of 56 nonlinear-domain HRV parameters were used in this study.

The dysglycemia and normal blood glucose data were compared using a *t*-test, and nine features with low significance (SI, AI, GI, LFN, SDANN1, MFDFA alpha1 Peak, MFDFA alpha2 Delta, MCVNN, and MFDFA alpha2 Asymmetry) and five features with multiple missing values (SDANN2, SDNNI2, SDANN5, SDNNI5, and ULF) were eliminated. A total of 77 features were used in this study.

## 4. Dysglycemia Prediction Model

In this study, data collected for 30 min (30 min long data) were used for each dysglycemia value of the prediction model based on the 77 HRV parameters extracted from one-hour-long data. Dysglycemia prediction was divided into three intervals based on the time of the blood glucose measurement: immediately after measurement, 15 min prior to measurement, and 30 min prior to measurement.

In this study, a one-dimensional (1D) convolutional neural network (CNN) model using residual blocks was used as the AI model for predicting dysglycemia. Residual block-based AI models have been widely used in the image processing field [29]. The dysglycemia prediction performances of ML and NN models, which are commonly used in ECG research, were compared during model selection. The results confirmed that the AI model used in this study exhibited outstanding performance in dysglycemia prediction despite using only ECG data.

**Figure 2:**
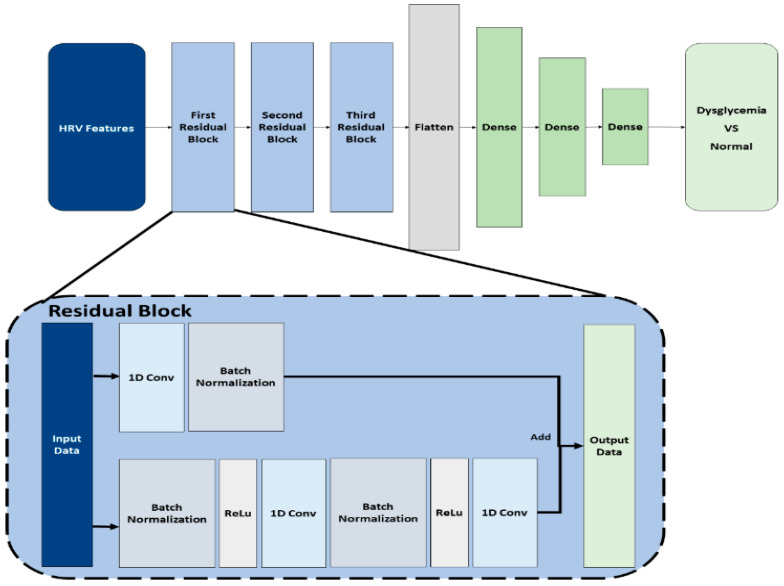
The architecture of the dysglycemia prediction model.

Figure 2 illustrates the architecture of the proposed dysglycemia prediction model. The HRV features extracted in the feature extraction process were divided into 30 min long data for each prediction segment and used as the model input. The upper part of the figure indicates that the input data passed through three residual blocks. The characteristics of these data were identified while minimizing their loss of weight. Subsequently, in the flattened layer, the data received from the residual blocks were structured into a 1D array to form a flattened matrix. Subsequently, as this flattened matrix passed through three dense layers, the number of data points in the matrix gradually decreased. Finally, the model predicted dysglycemia or normal blood glucose using the HRV features input to the model.

The lower part of the figure shows the structure of a residual block, which received data as input and passed them to the convolution layer at the bottom and the shortcut connection at the top. The convolution layer extracts information from the input data, whereas the shortcut connection transfers the weights of the input data to the computation result of the convolution layer. The output data were obtained by adding the computation result of the convolution layer to the input data transmitted from the shortcut connection. The weight transfer equation of the residual block can be expressed as follows:

H(x) = F(x) + x(1)

Data x input to the residual block in Equation (1) undergoes the convolutional operation in the residual block to become output data F(x). Subsequently, the data F(x) and the input data x are added to produce the final output data H(x). By adding blocks with simple calculations, the problem of vanishing or exploding weights can be solved as the neural network becomes deeper, thereby improving the performance.

The proposed dysglycemia prediction model was constructed to predict dysglycemia for each of the three aforementioned intervals using only the HRV features extracted from ECG data. This model compensates for the problems induced by conventional blood glucose measurement methods. Furthermore, the proposed AI model is capable of identifying as well as predicting dysglycemia.

## 5. Feature Selection

Feature selection was performed to optimize the prediction performance of the proposed AI model at each dysglycemia prediction point (at the time of blood glucose measurement, 15 min prior to measurement, and 30 min prior to measurement).

During feature selection, a subset of data deemed most important for the dysglycemia prediction model was identified from the 77 extracted parameters. The feature selection process was performed using the Shapley Additive Explanations (SHAP) library of Python [30]; SHAP is a game theoretic approach used for explaining the result of an AI model based on the Shapley value to extract the importance of each parameter for model prediction.

Table 3 lists the important features of the AI model when predicting dysglycemia at each prediction point; the top 10 features of the 77 features are listed.

We observed that MFDFA alpha2 Fluctuation in the nonlinear domain, which uses multifractal fluctuations, had the highest importance at the time point immediately after measuring the blood glucose, followed by CVSD in the time domain. At 15 min prior to the blood glucose measurement, SDNNa in the time domain had the highest importance, and DFA alpha1 in the nonlinear domain was also of high importance. At 30 min prior to the blood glucose measurement, PI in the nonlinear domain derived from the Poincaré plot was determined to be highly important. In summary, the HRV features in the nonlinear and time domains appeared to be highly important for predicting dysglycemia at each time point.

## 6. Results

### 6.1. Dysglycemia Prediction Results at Each Time Point

In this study, the parameters extracted during the feature selection process were listed in order of importance. These parameters were used as inputs for the proposed dysglycemia prediction model to verify the prediction results at each time point. Each time point was divided into three segments based on blood glucose measurement: immediately after blood glucose measurement, 15 min prior to measurement, and 30 min prior to measurement. The prediction accuracy of the dysglycemia prediction model was evaluated for each interval. Table 2 presents the input data used for the model; the cross-validation was performed in five folds. Subsequently, each result was subjected to statistical analysis using the Kruskal–Wallis test.

Table 4 summarizes the dysglycemia prediction results at each time point along with the statistical analysis results. On average, the prediction accuracy immediately after blood glucose measurement was 0.92 ± 0.013. The highest measured prediction accuracy was 0.93143, whereas the lowest prediction accuracy was 0.90095. In the case of 15 min prior to blood glucose measurement, the average prediction accuracy was 0.92 ± 0.013, with the highest and lowest prediction accuracies being 0.93905 and 0.90286, respectively. In the case of 30 min prior to blood glucose measurement, the average prediction accuracy was 0.92 ± 0.016, with the highest and lowest prediction accuracies being 0.93905 and 0.89333, respectively.

The Kruskal–Wallis test was conducted on the 5-fold prediction accuracy, excluding the average prediction accuracy in each segment. The results indicated a *p*-value of 0.8099, which exceeded the significance level of *p* > 0.05. This outcome indicates that no significant difference exists in the dysglycemia prediction accuracy in all three intervals, thereby validating the outstanding performance of the proposed prediction model.

### 6.2. Dysglycemia Prediction Results Based on Features

We evaluated the dysglycemia prediction results according to the number of features to determine the optimal number of features required for predicting blood glucose levels. Because the difference in prediction accuracy for each segment was not significant, we evaluated the results based on the prediction obtained 30 min prior to measurement, which exhibited the highest measurement range. Among the 77 features, the least important features were individually eliminated and the prediction accuracy for the results of the prediction model was compared. Subsequently, each result was subjected to statistical analysis using the Kruskal–Wallis test.

Table 5 summarizes the dysglycemia prediction results according to the number of features and statistical analysis. When all 77 features were used, the average prediction accuracy was 0.92 ± 0.016. The highest measured prediction accuracy was 0.93905, whereas the lowest prediction accuracy was 0.89333. When 30 features were used, the average prediction accuracy was 0.93 ± 0.007; here, the highest and lowest prediction accuracies were 0.93714 and 0.91810, respectively. The average prediction accuracy was 0.92 ± 0.004 when 20 features were used, with the highest and lowest prediction accuracies being 0.91985 and 0.90857, respectively. When 12 features were used, the average prediction accuracy was 0.905 ± 0.016, with the highest and lowest prediction accuracies being 0.92952 and 0.88571, respectively.

The Kruskal–Wallis test was conducted on the 5-fold prediction accuracy, excluding the average prediction accuracies of the various numbers of features. The results indicated a *p*-value of 0.0757, which exceeded the significance level of *p* > 0.05. We observed that if 11 features were used, the *p*-value changed to a significant level, thereby deteriorating the prediction performance. Therefore, the optimal number of features for predicting dysglycemia was determined to be 12 for the proposed dysglycemia prediction model.

Table 6 lists the 12 optimal features extracted for predicting dysglycemia. The features were grouped according to domain without considering the priority. The time domain included four features, whereas the frequency and nonlinear domains comprised two and six features, representing the lowest and highest number of features, respectively. Three of the features were based on entropy, and one feature was derived from MFDFA. Among them, two features were associated with the time domain, including the PI of a Poincaré plot, wherein the successive RR interval variation was less than zero, and the percentage of RR intervals of alternation segments.

Table 7 summarizes the dysglycemia prediction results obtained from the proposed model. The prediction results obtained using 12 features 30 min prior to measurement, which exhibited the highest prediction range, were verified using 5-fold cross-validation. The average accuracy was 0.905 ± 0.016, with the highest and lowest measured accuracies being 0.930 and 0.886, respectively. In the case of sensitivity, the average value was 0.875 ± 0.013, with the highest and lowest values being 0.886 and 0.852, respectively. The average specificity was 0.927 ± 0.021, with the highest and lowest values of specificity recorded as 0.961 and 0.907, respectively. The average value of precision was 0.898 ± 0.025, with the highest and lowest values of the measured precision being 0.942 and 0.883, respectively.

## 7. Discussion

The objective of this study was to predict dysglycemia before its occurrence to address dysglycemia-related issues in advance. The predictions were performed using an AI model and ECG data.

Recently, various studies have been conducted to predict and identify abnormal blood sugar levels using artificial intelligence models using electrocardiograms and various vital signs. Although similar studies to ours are being conducted, there are many limitations. Ling et al. [20] explored a non-invasive method to identify dysglycemia by using ECG data collected from 16 patients. In their study, the ECG data sampled at five-to-ten-minute intervals comprised 35 to 40 data points. They extracted four ECG features, including the total QTc and HRV, via preprocessing and used an extreme learning machine (ELM)-based neural network (NN) to identify dysglycemia. The results indicated a sensitivity of 78.00 and a specificity of 60.00, confirming that dysglycemia can be identified with high accuracy and speed using only ECG data. However, the scope of their study was limited to the identification of dysglycemia and did not include its prediction. Moreover, the sensitivity value was only 78.00, indicating that the model was not suitable for clinical applications. Zhu et al. [21] aimed to develop a smartphone-based platform for predicting and managing dysglycemia using an AI model. They used blood glucose measurements and vital signs (e.g., electrodermal activity (EDA), inter-beat interval (IBI), acceleration, and skin temperature) from 12 diabetic patients as inputs for the AI model. Additionally, they used an attention-based recurrent neural network (RNN) as the dysglycemia prediction model. The results indicated that IBI was the only predictor that exhibited a significant effect on both hypoglycemia and hyperglycemia prediction. The results further demonstrated that IBI or HRV can be useful biomarkers for predicting dysglycemia in patients with diabetes. The prediction results were verified at 15, 30, 45, and 60 min intervals, with the highest values observed at 15 min prior to measurement of hypoglycemia (accuracy: 98.03, sensitivity: 84.15, specificity: 98.72, and precision: 78.91) and hyperglycemia (accuracy: 96.75, sensitivity: 95.32, specificity: 96.95, and precision: 94.62). Subsequently, the prediction results gradually decreased. Although the aforementioned study confirmed that dysglycemia can be predicted up to 60 min in advance, it did not demonstrate that dysglycemia can be predicted using only ECG data because blood glucose measurements, EDA, acceleration, and skin temperature were used as inputs in addition to ECG data.

The data were collected using a wearable ECG monitoring patch from 83 patients with diabetes, who continuously need to manage their blood glucose levels. The participation criteria were strictly controlled, and patients who could potentially affect HRV and ECG data were excluded from this study. Consequently, data from 21 patients were used in the final analysis.

ECG data were used to address the issues originating from conventional methods of measuring blood glucose levels. The measured blood glucose data were used to classify patient data into dysglycemia and normal blood glucose. A total of 2624 ECG data points were selected, wherein 1042 and 62 data points corresponded to hyperglycemia and hypoglycemia, respectively, in the case of dysglycemia. In other words, the hyperglycemia data accounted for a larger proportion of the dysglycemia data. In the future, we intend to collect additional hypoglycemia data to equalize the proportions of hyperglycemia and hypoglycemia data when conducting experiments.

The HRV parameters were extracted from ECG data and used as inputs to the AI model for predicting dysglycemia. Additionally, the Pan–Tompkins algorithm was used to detect QRS complexes. A total of 91 HRV features were extracted during feature extraction by dividing the data into five-minute units. Subsequently, 14 features were eliminated via statistical analysis, and the remaining 77 features were used for the investigation.

The features selected from the extracted features were listed in order of importance for the prediction model to validate the results. The dysglycemia prediction results were subjected to statistical analysis by dividing the prediction into three segments, including immediately after blood glucose measurement, 15 min prior to measurement, and 30 min prior to measurement (Table 4). The analysis results indicated a *p*-value of 0.8099, which exceeded the significance level of *p* ≥ 0.05. This outcome implies that no significant difference exists in the dysglycemia prediction results at any of the aforementioned time points. Furthermore, it indicates that the proposed dysglycemia prediction AI model performs excellently at all time points.

To determine the optimal number of features required for predicting dysglycemia, 77 features were individually eliminated in the order of decreasing importance to verify the prediction accuracy (Table 5). Because the difference in prediction accuracy for each segment was not significant, we evaluated the dysglycemia prediction results based on the prediction 30 min prior to measurement, which exhibited the highest measurement range. The statistical analysis results indicate that the *p*-value was 0.0757 when 12 features were used, which exceeded the significance level of *p* ≥ 0.05. However, a significant difference was observed in the predicted results when 11 features were used, confirming that the optimal number of features required for predicting dysglycemia was 12.

Table 6 indicates the features that are important for dysglycemia predictions. Previous studies have reported that dysglycemia is related to the time domain of HRV [16,17,18]. The proposed dysglycemia prediction model concurred with this result, as the time-domain HRV features were most prevalent with six features.

In this study, a residual block-based 1D CNN model was used to predict dysglycemia, and its performance was compared with that of ML and NN models that are commonly used in ECG research.

Table 8 summarizes the comparison of the dysglycemia prediction results of the proposed residual block-based 1D CNN model, ML, and NN models. The comparison was performed using six ML models and three NN models. The six ML models comprised the support vector machine (SVM), logistic regression, decision tree, random forest, K-nearest neighbor (KNN), and AdaBoost. The three NN models included the artificial neural network (ANN), deep neural network (DNN), and long short-term memory (LSTM). We determined that the accuracy of the proposed model was up to 12% better than that of KNN, which exhibited an accuracy of 78%. Furthermore, the proposed model outperformed LSTM, which is widely used in time-series analyses, and DNN by 8% and 7%, respectively. Moreover, the proposed model outperformed the ML and NN models by up to 10% in terms of sensitivity (sensitivity of decision tree = 77.5%) and by up to 11% with respect to precision (precision of KNN and AdaBoost = 78%). Although the performance difference between the proposed model and the ML and NN models was the largest with respect to specificity, with a maximum difference of 15% (specificity of KNN = 77.5), the performance of DNN was slightly better than that of the proposed model by approximately 0.3%. According to the performance evaluation results obtained from the confusion matrix, the proposed model demonstrated better performance, confirming that the proposed residual-based 1D CNN model performed better than the ML and NN models in predicting dysglycemia.

Table 9 summarizes the comparison of the dysglycemia prediction results obtained in this study with those reported in other studies. The prediction in our study was performed using a residual block-based 1D CNN model based on 12 HRV features obtained from ECG data and by considering various prediction ranges. In this study, the dysglycemia prediction results at 30 min prior to measurement, which exhibited the highest prediction range, were as follows: accuracy = 90.5, sensitivity = 87.52, specificity = 92.74, and precision = 89.86.

Ling et al. [20], Cordeiro et al. [31], and Chiu et al. [18] extracted features from ECG data to identify dysglycemia using ML and NN models. Although these studies used only ECG data, they focused on identifying dysglycemia rather than predicting it. Ling et al. [20] reported a sensitivity of 78.00, specificity of 60.00, and gamma value (GV) of 70.80. Cordeiro et al. [31] reported a sensitivity of 87.57, specificity of 85.04, and area under curve (AUC) of 95.53. Chiu et al. [18] reported a sensitivity of 97, specificity of 96, AUC of 97. A significant difference of over 10% was observed between the results obtained in our study and those reported by Ling et al. [20]. However, the results reported by Cordeiro et al. [31] and Chiu et al. [18] demonstrated similar or superior performances compared to the results of this study.

Cichosz et al. [32] and Zhu et al. [21] used various vital signs, including ECG and CGM, to predict and identify dysglycemia. Cichosz et al. [32] employed both ECG and CGM in their study and developed an algorithm to identify dysglycemia, yielding the following results: sensitivity = 79.00; specificity = 99.00; and AUC = 99.00. In comparison with this study, the performance indicators in the study by Cichosz et al. [32] exhibited better results; however, the ECG and CGM data were used simultaneously and the prediction of dysglycemia was not explored.

Zhu et al. [21] used four vital signs, namely EDA, IBI, acceleration (ACC), and skin temperature (TEMP), along with CGM to predict dysglycemia via an attention-based RNN model at intervals of 15, 30, 45, and 60 min. The hyperglycemia prediction results were reported as follows: accuracy = 93.22, sensitivity = 91.25, specificity = 92.62, and precision = 90.51. The hypoglycemia prediction results were as follows: accuracy = 94.96, sensitivity = 76.08, specificity = 96.42, and precision = 65.65. In comparison with our results, these results indicate that the model reported by Zhu et al. [21] produced higher values in terms of accuracy, sensitivity, and precision during hyperglycemia prediction; in the case of hypoglycemia prediction, their model achieved higher accuracy and specificity. However, the differences in these values were not significant. Moreover, insufficient data on hypoglycemia may have affected the results of our study. Therefore, a more precise comparison is required in the future. Furthermore, Zhu et al. [21] used blood glucose values and various vital signs as inputs for the dysglycemia prediction model, whereas our study used only the HRV features extracted from ECG data as inputs.

Most of the existing studies on dysglycemia are limited to the identification of dysglycemia rather than its prediction. Moreover, considering that researchers have used various vital signs, including CGM data, the possibility of predicting dysglycemia using only ECG data has not been demonstrated thus far. Therefore, our study confirmed the possibility of using only ECG data to predict dysglycemia up to 30 min prior to measurement while addressing the issues originating during the measurement of blood glucose levels using conventional methods.

The dysglycemia prediction model of this study uses residual block, so it shows strong performance compared to other models even when using small data. In addition, since the structure is simple, it can yield fast prediction results, so it is possible to quickly analyze the collected data and make accurate predictions. It can also be used for healthcare purposes through glucose control in the general public without dysglycemia. When applied clinically, continuous glucose management of dysglycemia patients is possible with only an electrocardiogram, and it can prepare for acute dysglycemic shock. In addition, it can reduce the pain caused by existing glucose meters, medical waste generation, etc., and effectively solve problems such as measurement inaccuracy.

There are some limitations in our study. First, we did not obtain sufficient hypoglycemic data. The quantity of hyperglycemic data was greater than that of hypoglycemic data, and we plan to conduct the study again after obtaining sufficient data on hypoglycemic patients in the future. Second, we considered patients with cardiovascular diseases that affect ECG, such as the R-R interval, but ECG is very sensitive, so more factors should be considered. In addition, since it can be affected by diabetic autonomic neuropathy, we will conduct the study by considering this in more detail in future studies.

## 8. Conclusions

In this study, we predicted dysglycemia using only ECG data to enable the continuous management and prediction of blood glucose levels, which are both essential for patients experiencing hyperglycemia and hypoglycemia. The data were collected from patients with diabetes and ECG features were extracted for the prediction. A residual block-based 1D CNN model was used to predict dysglycemia. The model demonstrated superior prediction performance in comparison with conventional ML and NN models. The dysglycemia prediction results at each time point, including at the time of blood glucose measurement, 15 min prior to measurement, and 30 min prior to measurement did not exhibit significant differences compared with the blood glucose measurement values. This result confirmed that the proposed AI prediction model performs well in predicting dysglycemia at each time point. Furthermore, based on the analysis of 77 features considering their order of decreasing importance in terms of prediction accuracy, we determined that the optimal number of features for the prediction of dysglycemia was 12. The time-domain HRV features were maximum in number with six features.

The dysglycemia prediction results at 30 min prior to measurement, which exhibited the highest prediction range, were as follows: accuracy = 90.5, sensitivity = 87.52, specificity = 92.74, and precision = 89.86. No significant differences were observed in the dysglycemia prediction results between those reported in previous studies, wherein various vital signs and blood glucose values were used as inputs to the model, and the results of our study, which used only ECG data to predict dysglycemia. Therefore, the study findings validate that the proposed model can efficiently predict dysglycemia using only ECG data.

However, certain limitations were observed in our analysis. For instance, the data on hypoglycemia were insufficient during the experiment and a larger proportion of hyperglycemia data was employed in the analysis, which may have affected the results. In the future, we intend to investigate the potential of predicting dysglycemia using the same proportion of hyperglycemia and hypoglycemia data along with their classification. Furthermore, we intend to expand the range of blood glucose level prediction in future studies.

## Figures and Tables

**Table 1:** Patient information used in the study.

Total Number of Patients = 21			
Age		69 ± 8.6	
Gender (%), male		10 (47.6)	
DM (%)		21 (100)	
HbA1c (mmol/mol)		8.1 ± 1.26	
Measurement period (days)		9.8 ± 4.53	
Dysglycemia period (%)	Hypoglycemia	36.82 ± 26.36	1.74 ± 1.34
	Hyperglycemia		35.74 ± 26.76
Average Dysglycemia level (mg/dL)	Hypoglycemia	64.24 ± 2.08	
	Hyperglycemia	237.03 ± 34.19	
Normal blood glucose period (%)		63.17 ± 26.36	
Average normal blood glucose level (mg/dL)		128.75 ± 14.93	
Average blood glucose level (mg/dL)		172.14 ± 51.65	
Hypertension (%)		13 (61.9)	
Hyperlipidemia (%)		1 (4.76)	
Fatty liver (%)		1 (4.76)	
Gastric cancer (%)		1 (4.76)	
Insulin prescription (%)		6 (28.5)	
Metformin prescription (%)		8 (38)	
SGLT2 inhibitor prescription (%)		5 (23.8)	

**Table 2:** Selected ECG data.

Category		Range	Number of Data Points
Average Dysglycemia	Hypoglycemia	glucose < 70 mg/dL	62 (Train: 50; Test: 12)
	Hyperglycemia	glucose > 180 mg/dL	1042 (Train: 834; Test: 208)
Normal blood glucose		70 mg/dL ≤ glucose ≤ 180 mg/dL	1520 (Train: 1216; Test: 304)
Total			2624 (Train: 2100; Test: 524)

**Table 3:** Extraction of important features for predicting dysglycemia at each prediction point.

	Immediately After Blood Glucose Measurement	15 Min Prior to Measurement	30 Min Prior to Measurement
10 important features	MFDFA alpha2 Fluctuation	SDNNa	PI
	CVSD	DFA alpha1	SDSD
	MFDFA alpha1 Increment	SDNNd	CVNN
	Prc80NN	Prc80NN	MFDFA alpha2 Fluctuation
	pNN50	Prc20NN	PAS
	DFA_alpha1	MFDFA alpha1 Increment	RMSSD
	VHF	HFn	ApEn
	TP	VLF	ShanEn
	CMSEn	Ca	Prc80NN
	MFDFA alpha1 Width	SDRMSSD	HFn

**Table 4:** Dysglycemia prediction results at each time point and the statistical analysis results.

		Immediately After Blood Glucose Measurement	15 Min Prior to Measurement	30 Min Prior to Measurement	Kruskal Wallis Result
Prediction Accuracy	1 fold	0.91810	0.92762	0.89333	*p* > 0.05 *p*-value = 0.8099
	2 fold	0.90095	0.93905	0.92190	
	3 fold	0.91048	0.92000	0.93905	
	4 fold	0.93143	0.90286	0.92000	
	5 fold	0.93130	0.92939	0.92366	
	Average	0.92 ± 0.013	0.92 ± 0.013	0.92 ± 0.016	

**Table 5:** Dysglycemia prediction results according to the number of features and the statistical analysis results.

		77 Features	30 Features	20 Features	12 Features	Kruskal Wallis Result
Prediction Accuracy	1 fold	0.89333	0.92762	0.91429	0.91238	*p* > 0.05 *p*-value = 0.0757
	2 fold	0.92190	0.91810	0.91429	0.92952	
	3 fold	0.93905	0.93714	0.90857	0.90476	
	4 fold	0.92000	0.92952	0.91810	0.88571	
	5 fold	0.92366	0.92176	0.91985	0.89504	
	Average	0.92 ± 0.016	0.93 ± 0.007	0.92 ± 0.004	0.905 ± 0.016	

**Table 6:** Twelve optimal features used for dysglycemia predictions.

Domain	Features	Description
HRV time	SDSD	Standard deviation of successive RR interval differences
	CVNN	Standard deviation of RR intervals divided by the mean of RR intervals
	RMSSD	Root mean square of successive RR interval differences
	Prc80NN	The 80th percentile of RR intervals
HRV frequency	HFn	Normalized HF
	LFHF	Ratio of LF power to HF power
HRV nonlinear	MFDFA alpha2 Fluctuation	Estimation of signal fluctuation using multifractal detrended fluctuation analysis
	PAS	Percentage of RR intervals of alternation segments
	ApEn	Complexity estimation using approximate entropy
	ShanEn	Complexity estimation using Shannon entropy
	MSEn	Complexity estimation using multiscale entropy
	PI	Porta’s index of a Poincaré plot, in which successive RR interval variation is less than 0

**Table 7:** Dysglycemia prediction results of the model.

		Accuracy	Sensitivity	Specificity	Precision
Residual block based 1D CNN	1 fold	0.912	0.874	0.937	0.900
	2 fold	0.930	0.886	0.961	0.942
	3 fold	0.905	0.883	0.920	0.883
	4 fold	0.886	0.852	0.912	0.883
	5 fold	0.895	0.881	0.907	0.885
	Average	0.905 ± 0.016	0.875 ± 0.013	0.927 ± 0.021	0.898 ± 0.025

**Table 8:** Dysglycemia prediction results of the residual block-based 1D CNN, ML, and NN models.

	Accuracy	Sensitivity	Specificity	Precision
Residual block based 1D CNN	90.5	87.52	92.74	89.86
SVM	82.5	82	82	81.5
Logistic regression	80.8	80	80	80
Decision tree	79.1	77.5	78	78.5
Random forest	83.4	83	83	83
KNN	78	78.5	77.5	78
AdaBoost	78.7	78	78	78
ANN	81.9	81	81.5	81
DNN	84.3	82.5	93	85
LSTM	82.4	82	81.5	81.5

**Table 9:** Comparison of dysglycemia prediction results with those reported in other studies.

Study	Data	Method	Dysglycemia Prediction Range	Result
Our study	ECG (HRV)	Residual block based 1D CNN	Immediately after blood glucose, 15 min prior to, 30 min prior to measurement	Acc: 90.5, Sen: 87.52 Spec: 92.74, Pre: 89.86
Ling et al. [20]	ECG (HRV, QTC)	ELM Model	-	Sen: 78.00, Spec: 60.00, GV: 70.80
Cordeiro et al. [31]	ECG Feature 18	10-layer DNN	-	Sen: 87.57, Spec: 85.04, AUC: 95.53
Chiu et al. [18]	ECG Feature 25	Oc-SVM	-	Sen: 97, Spec: 96, AUC: 97
Cichosz et al. [32]	CGM, ECG (HRV)	In-house algorithm	-	Sen: 79, Spec: 99, AUC: 99
Zhu et al. [21]	CGM, Vital signs (EDA, IBI, ACC, TEMP)	Attention-based RNN	15 min prior to, 30 min prior to, 45 min prior, and 60 min prior to measurement	Hyperglycemia Acc: 93.22, Sen: 91.25 Spec: 92.62, Pre: 90.51
				Hypoglycemia Acc: 94.96, Sen: 76.08 Spec: 96.42, Pre: 65.65

## Data Availability

The datasets presented in this article are not readily available because the data are part of an ongoing study and due to ethical reasons.
